# Invasion of *Solanum tuberosum* L. by *Aspergillus terreus*: a microscopic and proteomics insight on pathogenicity

**DOI:** 10.1186/1756-0500-7-350

**Published:** 2014-06-10

**Authors:** Bengyella Louis, Sayanika Devi Waikhom, Pranab Roy, Pardeep Kumar Bhardwaj, Mohendro Wakambam Singh, Sharma K Chandradev, Narayan Chandra Talukdar

**Affiliations:** 1 Institute of Bioresources and Sustainable Development (IBSD), Takyelpat, Imphal 795001, Manipur, India; 2 Department of Biotechnology, The University of Burdwan, Golapbag More 713104, West Bengal, India; 3 Department of Biochemistry, University of Yaoundé I, BP812-Yaoundé, Yaoundé, Cameroon; 4 Department of Biotechnology, Haldia Institute of Technology, Haldia 721657, West Bengal, India; 5 Regional Centre of Institute of Bioresources and Sustainable Development (RCIBSD), Gangtok 737102, Sikkim, India

**Keywords:** Opposing accessory conidia, Proteome, Multipolar conidia germination, Stomata atropism, Lipoxygenase, Class I patatin, Scanning electron microscopy

## Abstract

**Background:**

*Aspergillus terreus* is one of the most harmful filamentous fungal pathogen of humans, animals and plants. Recently, researchers have discovered that *A. terreus* can cause foliar blight disease in potato (*Solanum tuberosum* L.). We used light and scanning electron microscopy, and performed proteomics analysis in an attempt to dissect the invasion process of *A. terreus* in this important crop.

**Results:**

Microscopic study revealed that invasion of leaf tissue is marked by rapid germination of *A. terreus* phialidic conidia (PC) by 4 h after inoculation. By 8 h after inoculation, primary germ tubes from PC differentiated into irregular protuberance, often displayed stomata atropism, and failed to penetrate via the epidermal cells. Colonization of leaf tissues was associated with high rate of production of accessory conidia (AC). These analyses showed the occurrence of a unique opposing pattern of AC, tissue-specific and produced on melanized colonizing hyphae during the infection of leaf tissue. A significant proteome change hallmarked by differential expression of class I patatin, lipoxygenase, catalase-peroxidase complex, and cysteine proteinase inhibitor were observed during tuber colonization. These proteins are often involved in signal transduction pathways and crosstalk in pathogenic responses.

**Conclusion:**

*A. terreus* abundantly produced AC and multipolar germinating PC to invade potato leaf tissue. Additionally, *A. terreus* differentially induced enzymes in potato tuber during colonization which facilitates rapid disease development.

## Background

The genus *Aspergillus*, a member of the phylum Ascomycota, includes over 185 known species [1]. *Aspergillus terreus* Thom (Deuteromycotina) belongs to the group of filamentous fungi which produces two types of asexual conidia viz., 1) the ultra-small size phialidic conidia (PC), mainly produced at the tips of conidiophores, and 2) the globose-hyalinated accessory conidia (AC), which emerges laterally from hyphae. Although *A. terreus* is beneficial for industrial production of lavastatin, gliotoxin and bioethanol [2], the pathogen causes severe damages in agriculture and human health [3]. Disturbingly, there is prediction that 4% of all patients who die in hospitals die of invasive aspergillosis [4]. *A. terreus* causes severe loss to important crops worldwide, and destroying over 125 million tons of rice (*Oryza sativa* L.), wheat (*Triticum aestivum*), potato (*Solanum tuberosum* L.), maize (*Zea mays*) and soyabean (*Glycine max* L*.*) every year [3,5].

Despite the vast studies on invasive aspergillosis [6-9], the mode of colonization of plant host by *Aspergillus* species is poorly understood. Nonetheless, it has been proposed that injuries on plant tissues are prerequisite for successful colonization [10,11]. At the farm level, host genotype, soil type, drought conditions and high level insect activities are important factors that determine the dissemination and development of *Aspergillus* diseases [12]. On a putative host, *A. terreus* produces toxic metabolites such as territrem A, territrem B and territrem C [13], which enhance pathogenicity. Recently, *A. terreus* is shown to cause root rot diseases in wheat and *Lolium* species [14]. In potato, foliar blight caused by *A. terreus* amounts to 30-60% of the total leaf surface [15,16], but the infection process is not elucidated. Therefore, we set as objective to study the infection process of potato by *A. terreus*.

## Results and discussion

### Phylogenetic placement of the studied strain

**Figure 1:**
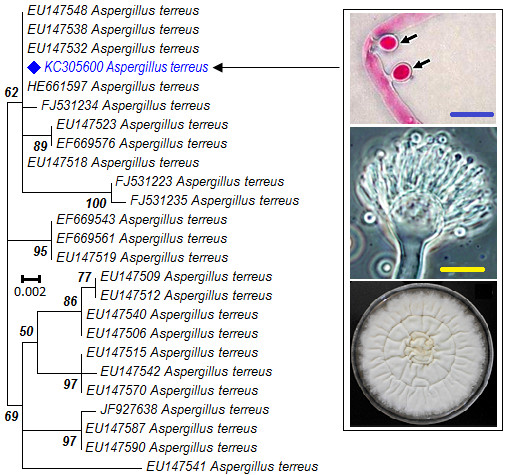
**Molecular phylogenetic analysis by Maximum likelihood method (ML) based on the K2 + G substitution model.** AIC is 1953.78, BIC is 2311.02; the highest log likelihood is −953.45 and bootstrap values ≥ 50% from 1000 iterations are shown. Blue highlighted strain of *A. terreus* (GenBank® accession number KC305600) causes foliar necrosis of potato. The ML analysis was performed in MEGA 6 [34]. Morphological characteristics of globular accessory conidia indicated by arrows is stained with Rose Bengal, broom-like conidiophore and colony on PDA, scale bar = 20 μm and magnification = 1000X.

**Figure 2:**
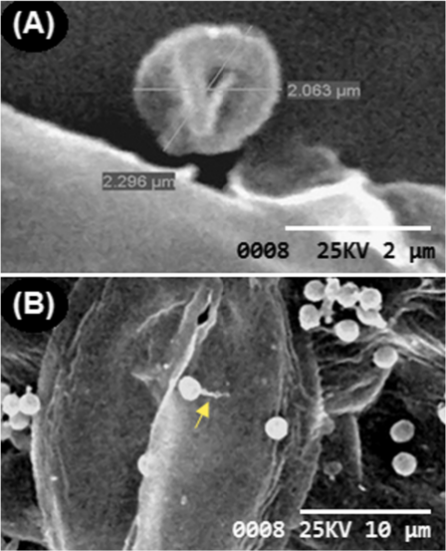
**Scanning electron microscopy micrographs showing the pattern of colonization by *A. terreus *(GenBank® accession number KC305600) phialidic conidia on potato cv. Kufri Jyoti leaf. (A)** Dormant phialidic conidia by 2 h after inoculation at 4000X. **(B)** Stomata atropism by germinating conidia at 8 h after inoculation at 3000X.

By comparing at the level of *calmodulin* (*Cmd*) locus, our strain of *A. terreus* (GenBank® accession number KC305600) with reference strains available at NCBI nucleotide data base, a total of 109 patterns out of a total of 729 sites were found and 670 sites were without single nucleotide polymorphism (92.48%). Based on the *Cmd* locus, our strain of *A. terreus* (GenBank® accession number KC305600) showed 98% identity with *A. terreus* (GenBank® accession number EU147532) but failed to cluster with other strains (Figure 1). Closely related strains to *A. terreus* (GenBank® accession number KC305600) were all singletons (or unclustered strains) suggesting divergent evolution (Figure 1). Further information associated with phylogenetic placement of the studied *A. terreus* is available in Dryad Digital Respository as http://dx.doi.org/10.5061/dryad.590j0. This strain (GenBank® accession number KC305600), hereinafter designated as *A. terreus,* produced small aseptate phialidic conidia (2.1–2.3 μm diameter), with 2–3 deep grooves that tapered into a hornlike projection (Figure 2A). Clinical strain previously described based on scanning electron microscopy (SEM) micrograph [6] had no hornlike projection and no deep grooves.

### The infection process

**Figure 3:**
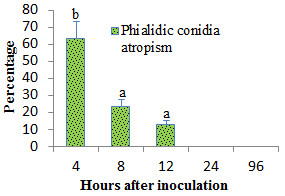
**The rate of phialidic conidia undergoing stomata atropism during the invasion of potato cv. Kufri Jyoti leaf.** Bars represent standard errors of the mean values and ^a, b^denote mean treatments that are significantly different according to Tukey’s test at *P < 0.05*.

The epidemiology of *A. terreus* related diseases in crops are well documented [10,11,14,15], but, the infection process is unreported. Importantly, it was shown that primary infection is enhanced by drought stress in peanut (*Arachis hypogaea* L.) leaf canopy and injuries in stored grains [10,11,17]. Using detached leaf technique, we dissected the infection process on potato cv. Kufri Jyoti from which the virulent *A. terreus* was isolated from the field. It was observed that phialidic conidia (PC) stayed inert on potato leaf for 2 h after inoculation (Figure 2A). By 4 h after inoculation, 63.33% (*F = 1353.21, P < 0.05*) of all germinated PC moved away from the stomata. Noteworthy, by 8 h after inoculation, 23.33% (*F = 1353.21; P < 0.05*) of all germinated PC moved away from the stomata, thus, displayed stomata atropism (Figure 3). Stomata atropism is the inability of a germinating fungal conidium to penetrate via the stomata pore.

**Figure 4:**
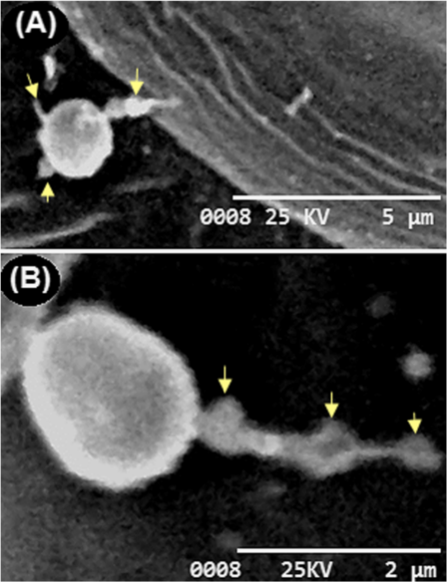
**Scanning electron microscopy (SEM) micrographs showing the pattern of colonization by *A. terreus *phialidic conidia on potato cv. Kufri Jyoti leaf. (A)** Phialidic conidia produced multipolar germ tubes by 8 h after inoculation, imaged at 3000X. **(B)** Differentiation of germ tubes into irregular protuberance (indicated by arrows) by 24 h after inoculation, imaged at 5000X.

**Figure 5:**
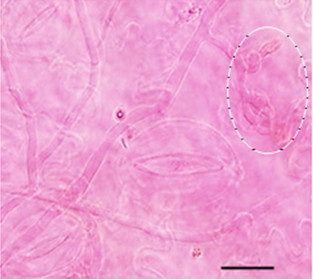
**Light microscopy micrograph by 72 h after inoculation of *A. terreus *showing the differentiation of colonizing hyphae tips (encircled) on potato cv. Kufri Jyoti leaf.** No direct penetration of the leaf tissue was detected. Staining was performed using 4,5,6,7-tetrachloro-2,4,5,7-tetraiodofluorescein at 1000X, scale bar = 20 μm.

**Figure 6:**
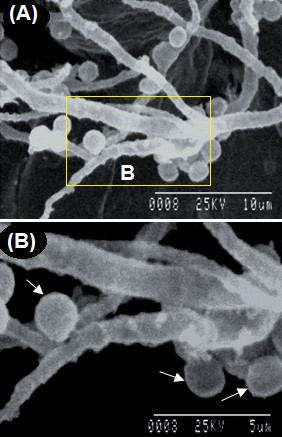
**Scanning electron microscopy (SEM) micrographs showing the abundant production of *A. terreus *accessory conidia (indicated by arrows) on potato cv. Kufri Jyoti leaf by 96 h after inoculation. (A)** Ramification of AC with hyphae at 1500X, scale bar = 10 μm. **(B)** Close-up of Figure 5A at 2800X, scale bar 5 μm.

Remarkably, multipolar germinated PC were detected by 8 h after inoculation, and these results showed *A. terreus* PC colonized potato leaf tissue in multi-directions (Figure 4A) leading to the development of foliar blight (Additional file 1: Figure S1). Importantly, irregular protuberance (IP) was detected on the colonizing germ tubes by 8 h after inoculation on leaf tissue (Figure 4A). By 24 h after inoculation of leaf tissue, the hyphae spread rapidly and the interconnected IP from colonizing germ tubes became predominant, averaging 0.2–0.5 μm in diameter (Figure 4B). The exact role of this IP is not known. We suggest that it may play a key role in keeping the germinated PC adhered on potato leaf tissue. *A. terreus* is a rapid colonizer and by 72 h after inoculation, colonizing hyphae had differentiated, and formed networks of hyphae that cover the leaf tissue. Nevertheless, no direct leaf tissue penetration was observed (Figure 5). At 96 h of infection, *A. terreus* profusely sporulated (Figure 6A: Additional file 1: Figure S1A) on leaf tissue. It is worth mentioning that fungal spores of phytopathogenic fungi are important virulence factor [18]. The direct consequence of rapid growth and sporulation was marked by the destruction of leaf epidermal cells, formation of white mycelia patches on the abaxial and adaxial leaf surface (Additional file 1: Figure S1B). Throughout the experimentation, there was no instance of direct penetration of the leaf tissue by *A. terreus* PC (Figure 5). Based on SEM analysis, *A. terreus* was shown to produce appressorium during interaction with *Sclerotinia sclerotiorum*[19]. In this study, no appressorial structure was observed on potato leaf tissue. Thus, the data revealed that *A. terreus* PC preferentially colonized potato leaf superficially.

**Figure 7:**
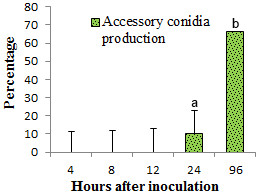
**The rate of production of *A. terreus *accessory conidia on potato cv. Kufri Jyoti leaf.** Bars represent standard errors of the mean values and ^a, b^ denotes mean treatments that are significantly different according to Tukey’s test at *P < 0.05*.

**Figure 8:**
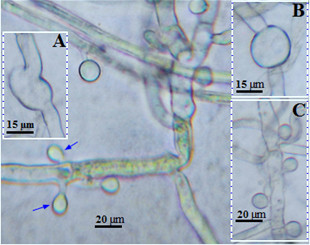
**Phase contrast micrograph depicting morphological divergence in *A. terreus *accessory conidia produced during colonization of potato cv. Kufri Jyoti leaf by 96 h after inoculation. (A)** The pathogen hyphae forms interconnected protuberance either club-shaped at 1000X, **(B)** ellipsoidal at 1000X, **(C)** lateral arising accessory conidia at 800X and opposing accessory conidia at 1000X (indicated by arrows).

Accessory conidia (AC) is an important virulence factor in *A. terreus* pathogenicity [7,8,20]. Nevertheless, the exact role played by AC in aspergillosis is unknown [7-9,20]. Additionally, very little is known whether *A. terreus* produces AC on putative plant hosts. In this study, it was observed that *A. terreus* abundantly produced AC during the infection process on potato cv. Kufri Jyoti leaf (Figure 6). By 96 h after leaf inoculation, the rate of production of AC was significantly high (Figures 6 and 7). It is worth noting that, a maximum number of AC was observed at 66.67% (*F = 3967.31, P < 0.05*) per 20 μm^2^ of colonized leaf tissue by 96 h after inoculation (Figure 7). In most instances after 24 h of leaf inoculation, abundant production of AC and hyphae networking masked our ability to follow-up germinated PC exhibiting stomata atropism. By using light microscopy, we observed that the IP showed variations in forms, from ellipsoidal to club-shape and often associated with AC by 96 h after inoculation of leaf tissue (Figure 8A, B).

**Figure 9:**
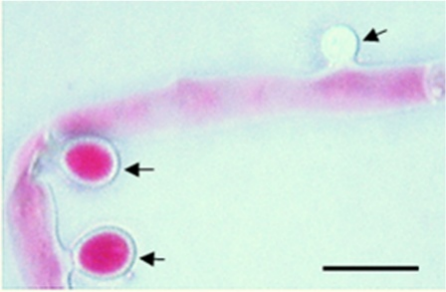
**Typical pattern of *A. terreus *lateral globose accessory conidia (tagged with arrows) occurring in an alternating-thorn-like pattern on potato cv. Kufri Pukraj leaf.** This pattern of accessory conidia was also observed on tuber slices of potato cv. Pukraj and potato cv. Kufri Jyoti. Staining was performed using 4,5,6,7-tetrachloro-2,4,5,7-tetraiodofluorescein at 1000X, scale bar = 20 μm.

The usual occurrence pattern of AC on hyphae is an alternating-thorn-like distribution (Figure 8C), analogous to previous observations [6-9,15,20]. It is interesting to remark that, beside the alternating-thorn-like arrangement of AC, it was also found that melanized colonizing hyphae produced opposing AC on potato cv. Kufri Jyoti leaf (Figure 9). Tuber slices of potato cv. Kufri Jyoti, and the leaf and tuber slices of potato cv. Kufri Pukraj were used to check the occurrence of this unique opposing AC pattern. The results were only positive on potato cv. Kufri Jyoti leaf, signifying specific host–tissue signal is responsible for the pattern of formation of opposing AC observed only on potato cv. Kufri Jyoti leaf.

Strikingly, using clinical isolates, Deak *et al.*[20] reported that AC morphology varies among strains and remains fairly consistent for any given strain. In contrast; it is found herein that opposing AC not reported before is produced by *A. terreus* during invasion on potato cv. Kufri Jyoti leaf tissue (Figure 8). Another question arises as to why opposing AC developed during potato leaf colonization are not observed on the potato tuber? Wilson *et al.*[21] suggested that a pathogenic fungus could receive morphological and chemical signals from host plant which are direct consequence of fungal invasion. According to Lass-Flörl *et al.*[22], host characteristics as well as inoculum size could affect *A. terreus* virulence. Based on these previous studies [21,22], we concluded that the opposing AC is produced as a function of specific host tissue signal.

*A. terreus* AC was demonstrated to have significant amount of metabolic activity [20]. Thus, AC ultimately excretes waste metabolic products which might be toxic to the host. Often, fungi and fungal spores are able to colonize and infiltrate into the matrices of agricultural crops and produce mycotoxins causing damage [13,23]. *A. terreus* generally produces toxic metabolites on host [4,5,9]. As shown (Figure 5), *A. terreus* spores (i.e. PC and AC) does not penetrate the leaf tissue during invasion. However, *A. terreus* abundantly produced AC during colonization (Figures 6, 7, 8 and 9). Collectively, there is likelihood that waste metabolites produced from AC might negatively affect the host defense leading to the development of disease. *A. terreus* is often explored as a bioagent for pest control [19,24]. Nevertheless, *A. terreus* is an efficient cellulase producer [25,26]. Cellulase is a key virulent factor for most phytopathogenic fungi [26,27]. We suggest that the foliar disease (Additional file 1: Figure S1B) akin to previous study [15], might be due to cellulolytic activity, and the discharge of toxic metabolic waste from the propagation of *A. terreus* since no direct penetration was observed (Figure 5). Inoculum size and host characteristics are also suggested to affect *A. terreus* virulence in animal models [22]. Additionally, because the amount of AC increased during the infection process (Figures 6 and 8), it can be concluded that *A. terreus* inoculum equally increases during colonization. Also, the abundant production of AC colonization indicates it plays a key role in *A. terreus* pathogenicity on potato leaf. Elsewhere, it was reported that the production of AC induces heightened inflammatory responses in a pulmonary model, and also participates in interaction with macrophages [7,28]. As shown in this study, abundant production of AC is associated with successful colonization of potato leaf.

### Analysis of proteome changes during colonization

**Figure 10:**
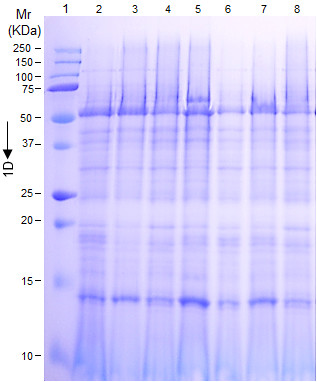
**SDS-polyacrylamide gel electrophoresis showing insignificant changes in potato cv. Kufri Jyoti crude leaf proteins during interaction with *A. terreus*.** Lane 1 is Precision Plus Protein^TM^ WesternC^TM^ standards (Bio-Rad, Hercules, CA, USA). Lane 2- Unchallenged potato leaf protein which stayed constant at all the experimental time points. Lane 3, 4, 5, 6, 7 and 8 are crude proteins at 2, 4, 8, 24, 72 and 96 h respectively, after inoculation of *A. terreus*. Gels were stained with Coomassie Blue R250.

**Figure 11:**
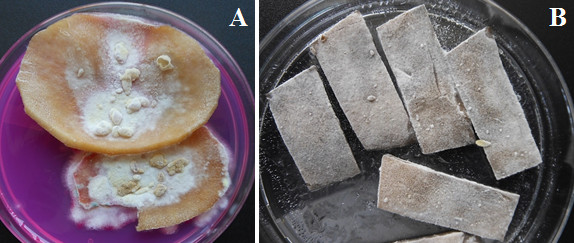
**Qualitative rate of colonization of aseptic potato slices by *A. terreus *GenBank® KC305600. (A)** 48 h after inoculation. **(B)** 96 h after inoculation marked sporulation of *A. terreus* which changes the appearance of tuber slices.

In order to understand the mechanism by which *A. terreus* interact with potato host, one dimensional SDS-polyacrylamide gel electrophoresis (1-D) was used. The changes in leaf and tuber proteins of potato cv. Kufri Jyoti were studied. No significant change in leaf proteins at different time points was observed (Figure 10), and because of this, we focused on significant proteome changes in tuber slices. *A. terreus* rapidly invaded potato cv. Kufri Jyoti tuber slices rendering it difficult to quantify the rate of colonization (Figure 11). Qualitatively, *A. terreus* produced an effuse whitish colony (of average diameter 20 mm) by 48 h after inoculation on potato slices (Figure 11A). By 96 h after inoculation, *A. terreus* completely colonized potato cv. Kufri Jyoti tuber slices (of sizes 6 cm × 0.75 mm × 0.75 mm), and produced a brownish-white appearance (Figure 11B).

**Figure 12:**
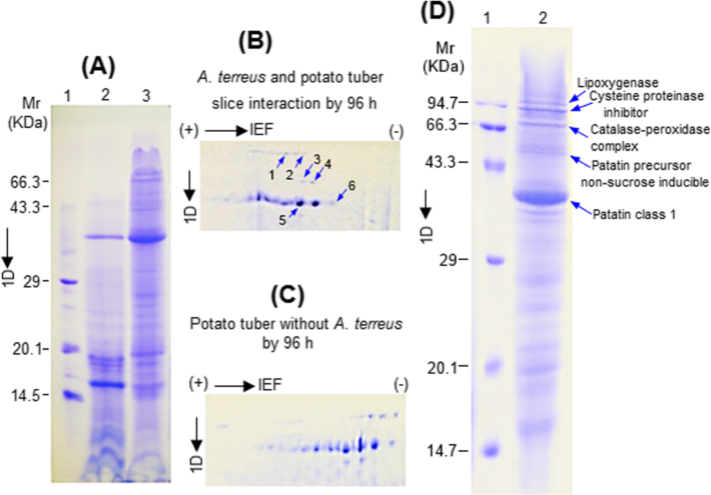
**SDS-polyacrylamide gel electrophoresis showing proteome changes during interaction of *A. terreus *with potato tuber slice by 96 h. (A)** Crude 20 μg proteins was separated by 1-D on a 15% gel; lane 1, 2 and 3 are molecular weight marker, potato tuber proteins without *A. terreus* and potato tuber inoculated with *A. terreus*, respectively. 2-D images of crude proteins showing region of interest: Isoelectric focusing (IEF) was performed with 140 μg of proteins on an IPG strip (pH 4–7, 7 cm) followed by separation on a 15% gel; **(B)** inoculated tuber and **(C)** unchallenged tuber. **(D)** 1-D profile for identified induced proteins during colonization of potato tuber slice by 96 h and all gels were stained with Coomassie Blue R250.

We observed a significant proteome change by 96 h only, after inoculation of *A. terreus* on tuber slices using 1-D analysis (Figure 12A). Additionally, crude proteins obtained at 96 h after inoculation of tuber was further separated by two dimensional SDS-polyacrylamide gel electrophoresis (2-D) for high resolution (Figure 12B, C). Herein, only proteins from *A. terreus* interaction with potato tuber were identified and discussed (Figure 12D: Table 1). Based on matrix-assisted laser desorption/ionization time-of-flight/time-of-flight tandem mass spectrometry (MALDI-TOF/TOF MS/MS), spot-1 was identified as potato lipoxygenase (pLOX). It is worth mentioning that LOX is an enzyme whose catalytic activity depends on a non-heme iron prosthetic group at the catalytic site. LOX plays a crucial role in the production of reactive oxygen species (ROS) during pathogen attack [29]. Spot-2 was identified as cysteine proteinase inhibitor (pCPI). Generally, pCPI plays multifarious roles which include degradation of storage proteins, turnover of stressed or damaged proteins, and programmed cell death associated with hypersensitive reaction in the case of pathogen attack [30,31]. Spot-3 was identified as a catalase-peroxidase (CATP) complex secreted by *A. terreus* during infection. This enzymatic complex generally scavenges hydrogen peroxides and ROS [31]. The production of pLOX and CATP complex by potato and *A. terreus*, respectively; strongly suggests functional interactive antagonism during invasion. Spot-4 was identified as patatin precursor, non-sucrose-inducible (PPSI) of potato. Spot-5 was identified as class I patatin of potato having lipase activity. Spot-6 was identified as a transporter protein expressed by *A. terreus*, as a result, this peptide spot did not match with any peptide spot on control tuber slice (Figure 12B, C). Noteworthy, most of the conspicuous untagged spots on the 2-D gels (Figure 12B, C) were identified as fragments of patatin (data not shown). Overall, results suggest most of the identified differentially expressed proteins on colonized potato tuber had enzymatic activities as well as defense related putative functions (Figure 12D: Table 1). Worth noting, results from MALDI-TOF/TOF MS/MS analysis produced some variations in experimental and theoretical *pI* and Mr values (Table 1). Such variations are common in mass peptide fingerprinting analysis [18,32,33]. It is suggested that variations can be due to post translational modifications such as ubiquitination, sumoylation, glycosylation, alternative splicing, endoproteolytic cleavage, and ecological niche of the host [18,32,33]. It might be possible that proteolytic degradation occurred in this study based on the evidence that pCPI was up-regulated (Table 1).

**Table 1:** **Identified differentially expressed proteins following interaction of *Aspergillus terreus *** **with potato tuber**

^**a**^**Sp**	^**b**^**Diff. exp.**	**Protein identity**	**Accession**	**Source**	^**c**^**Exp. pI/**^**d**^**Thr. pI**	^**e**^**Sc.**	^**f**^**Cov.**	^**g**^**F.exp.**	**Putative function**
1	Up-regulated	Lipoxygenase	O22507	*Solanum tuberosum* L.	6.31/6.49	100	50	2.1	Defense/disease
2	Up-regulated	Cysteine proteinase inhibitor	S38742	*Solanum tuberosum* L.	7.69/7.78	100	56	2.9	Defense/disease
3	Non-matching	Catalase-peroxidase	Q96VT4	*Aspergillus nidulans*	6.06/5.89	62	45	-	Defense/disease
4	Up-regulated	Patatin precursor non-sucrose-inducible	S51596	*Solanum tuberosum* L.	7.69/7.90	108	70	1.8	Metabolism
5	Up-regulated	Patatin class 1	T07592	*Solanum tuberosum* L.	5.32/5.18	60	45	1.5	Metabolism
6	Non-matching	Transport protein USO1	Gi/28881127	*Neurospora crassa*	5.17/5.07	57	30	-	Transport

^a^Spot number corresponds to spots in Figure 11, ^b^Differential protein expression, ^c^Experimental isoelectric point (*pI*), ^d^Theoretical *pI*, ^e^Score of protein reported by MASCOT at *P < 0.05*, ^f^Amino acid sequence coverage, and ^g^Fold change in expression of peptide spot.

As a whole, how pLOX, pCPI, CATP and PPSI interacts during *A. terreus* invasion on potato tuber is not known and requires further investigation. Nevertheless, it might be possible that once potato tuber senses *A. terreus*; class I patatin having lipase activity is differentially up-regulated, which mobilizes lipid reserves. This might trigger the induction of pLOX which performs lipid peroxidation and produces reactive oxygen species (ROS). These ROS can negatively affect the fungal development [31]. Nonetheless, *A. terreus* could circumvent pLOX ROS-mediated defence by overexpressing CATP to neutralize ROS and arrest host programmed cell death. Therefore, a type of functional antagonism is observed between pLOX and CATP. Nonetheless, the neutralizing effect possibly triggers potato tubers to express pCPI to carry out a global proteolytic reaction for all stressed proteins at the infection site. Although the potato tuber might appear to defend itself by expressing a plethora of defence-related enzymes, abundant expression of pLOX appears crucial since it can inhibit fungal development [34,35]. An interesting approach to study plant–*A. terreus*– human interactions was conceived, and brought insights on the host shifting virulence of *A. terreus*[6]. Lass-Flörl *et al.*[9] also showed that potted plants infected with *A. terreus* present near patients in a hospital, latter on caused lethal infections in nine patients subjected to myloblative chemotherapy. This shows *A. terreus* is a harmful pathogen [3,8,16] and should be studied using plant and animal models in order to understand its mechanism of colonization.

## Conclusion

To conclude, abundant production of accessory conidia on potato leaf and differential expression of enzymes on potato tuber slices are crucial for successful colonization of potato crop. Our data contributes towards *A. terreus* intractable pathogenicity of *A. terreus* in host plants.

## Methods

### Microorganism, plant growth and interactions analysis

The type isolate *A. terreus* (GenBank® as accessions KC305600) which caused foliar blight of potato was used [15]. Sequence sets from GenBank were screened and ambiguous sequences were eliminated using ElimDupes server (available at http://hcv.lanl.gov/content/sequence/ELIMDUPES/elimdupes.html). Sequence alignment was performed using ClustalW. Best substitution model parameters were determined based on Akaike Information Criterion, corrected (AICc) and Bayesian Information Criterion (BIC). The evolutionary history was inferred using the Maximum Likelihood (ML) method in MEGA6 software [36]**]**. The strength of the internal branches formed in the ML tree was statistically tested by 1000 bootstrap replications.

Potato cv. Kufri Jyoti and potato cv. Kufri Pukraj were grown in 7 L capacity trays containing autoclaved soil in a plant growth chamber (U-CON250, Labtech Co., Ltd, Danihan, India) at 20°C, and at 80% relative humidity (RH). The soil was derived from a blend of rice husk vermicompost and sand (1:2% w/w). The soil was amended with 1 g of Nitrogen-Phosphorous-Potassium (N-P-K; 1:1:1% w/w) fertilizer after one week of sprouting. The average light intensity was 180 μmolm^−2^ s^−1^ with photoperiod of 16 h light and 8 h darkness. Potato cv. Kufri Jyoti is grown in India and it shows salient resistance features to *Phytophthora infestans* (http://nhb.gov.in/vegetable/potato/pot013.pdf). *A. terreus* was cultured on potato dextrose agar (PDA) and incubated at 25°C for 1 week. To prepare PC inoculum, we gently scrapped colonies with sterile forceps loops in 4 ml sterile water. PC was pelleted by centrifugation at 13,000 g for 2 min and adjusted to 10^6^ cell/ml in sterile water using a haemocytometer (Swastik Scientific Co., Germany). The inoculum was stored at 4°C and used throughout the experiment unless otherwise mentioned.

A time course SEM analysis was performed to decipher the superficial interaction of *A. terreus* with potato cv. Kufri Jyoti leaf. Inoculated leaf disc (15 mm diameter) were incubated for 2, 4, 8, 12, 24 and 96 h after inoculation at 20°C and at near 100% RH on sterile water-moist filter paper. Leaf discs were treated for SEM as previously described [37]. Gold coating was performed with IB_2_-gold coater (HiTachi® Japan) in an argon atmosphere. The samples were scanned with S-530 SEM (HiTachi® Japan) at 25 KV accelerating voltage. Three samples were observed per treatment for a total of 3 biological replicates. The experiments were performed in a full randomized block design. Phialidic conidia stomata atropism was evaluated in parallel with the amount of AC produced every 20 μm^2^ per leaf tissue.

For light microscopy, leaf tissue was stained with freshly prepared 0.3% of 4,5,6,7-tetrachloro-2,4,5,7-tetraiodofluorescein (Sigma®, USA) as earlier described [15]. Because of the occurrence of the unique opposing AC pattern on potato cv. Kufri Jyoti leaf, we used tuber slices of same cultivar to test whether the opposing AC pattern was tissue-specific. We also used potato cv. Kufri Pukraj tuber slices and leaf to test the occurrence of opposing AC. The observation was performed with a microscope coupled with DP7M5.0.0.5 software and an Olympus DP70 camera (Olympus BX61®, USA). All the treatments were performed in triplicates. All data were subjected to One-way ANOVA associated with Tukey’s HSD post-hoc test to determine the mean significant differences between treatments at 5% level of significance.

### Protein extraction and quantification

Motivated by the difference in the pattern of production of AC on the leaf and tuber slice, we checked for the changes in proteome in these tissues during interaction. Disease-free tuber slices of potato cv. Kufri Jyoti (6 cm × 0.75 mm × 0.75 mm thickness) were established with a microtome knife, and treated with 500 mg/l of chloramphenicol for 6 min to avoid bacterial infection. The slices were placed in sterile petri plates and spotted with 50 μl of PC inoculum*.* Control potato slices were inoculated with 50 μl sterile water only. The petri plates were sealed with parafilm paper and incubated at 20°C under 8 h photoperiod. Potato cv. Kufri Jyoti plants were treated with the same concentration of inoculum in the growth chamber. Control plants were spread with sterile water only. At 2, 4, 8, 12, 24 and 96 h, 1 g of leaves and tuber slices for each experimental setup was collected in a randomized block manner and crushed in a pre-chilled mortar and pestle in 10 mM CaCl_2_ solution containing 0.25% Triton-X-114 (Sigma®, USA) and 1% of dithiothreitol (DTT, Sigma®, USA). The homogenate was centrifuged at 10,000 g for 10 min at 4°C and supernatant was retained. 150 μl of pre-chilled precipitation solution (consisting of 50 ml of 100% trichloroacetic acid and 50 ml of 100% acetone) was added to 1 ml of supernatant. The mixture was incubated overnight at -20°C for slow precipitation of proteins. The sample was pelleted at 13,000 g for 10 min and pellet was washed with ReadyPrep™ 2-D cleanup Kit® (Bio-Rad, Hercules, CA, USA) following the manufacturer instructions. Pellets were suspended in ReadyPrep^TM^ rehydration buffer consisting of 8 M urea, 2% CHAPS, 50 mM DTT, 0.2% (w/v) Bio-Lyte® 3/10 ampholytes, and traces of Bromophenol Blue (Bio-Rad, Hercules, CA, USA). 10 μl of protein aliquots was quantified by the dye-binding method [38] spectrophotometrically at 595 nm using bovine serum albumin to generate a standard curve.

### SDS-polyacrylamide gel electrophoresis, peptide fingerprinting and database searching

One dimensional SDS-polyacrylamide gel electrophoresis (1-D) was performed as earlier described on a 15% SDS-polyacrylamide gel [39]. 2-D was performed as follows. Briefly, immobilized pH gel (pH 4–7 IPG, 7 cm, Bio-Rad®, USA) were rehydrated passively with 140 μg of proteins for 16 h at room temperature. Isoelectric focusing (IEF) was performed using a default rapid ramp option in Protean®i12 IEF Cell (Bio-Rad®, USA) at 20°C. IPG strips were equilibrated twice for 30 min in equilibration buffer I (50 mM Tris–HCl pH 8.8, 6.5 M urea, 30% (v/v) glycerol and traces of Bromophenol Blue, 2% DTT) and equilibration buffer II (50 mM Tris–HCl pH 8.8, 2.5% iodoacetamide), respectively. The second dimensional separation was performed on a 15% SDS-polyacrylamide gel. The run was performed at 120 V in a 1X Tris-glycine-SDS, pH 8.3 (25 mM Tris–HCl, 200 mM Glycine, 0.1% SDS) running buffer in PowerPac^TM^ Basic 300 V system (Bio-Rad®, USA) as described earlier [39]. Gels were stained with 0.30% Coomassie Brillant Blue R250 (SRL, Mumbai, India) solution overnight. Destaining was performed in a solution containing 50% methanol and 10% acetic acid until visible bands or spots were seen. The gels were scanned using VersaDoc^TM^ 300 (Bio-Rad®, USA). For 2-D images, region of interest was analysed using TotalLab Progenesis SameSpot 4.1 for spot detection and background subtraction. Peptide spots were subjected to Anova-test to check the significance of expression at *P < 0.05*. Reproducible and differentially expressed peptide spots in 2-D analysis with molecular weight corresponding to induced and up-regulated protein bands observed in 1-D analysis were excised for downstream analysis. The entire 2-D and 1-D experiment was repeated with three biological replicates. Each protein sample per biological replicate was resolved by 1-D and 2-D at least three times.

Manually excised peptide bands and spots were subjected to trypsin digestion and elution as earlier described [40]. 0.45 μl of digested protein solution was sandwiched in 5 mg/ml α-cyano-4-hydroxy-cinnamic acid (diluted in 0.1% triflouroacetic acid, 50% acetonitrile) on a matrix assisted laser desorption/ionization (MALDI) target plate (Applied Biosystems, Vernon Hills, IL, USA). MALDI-TOF/TOF MS/MS was performed in SCIEX4800 MALDI TOF-TOF proteomics (Applied Biosystems, Vernon Hills, IL, USA) at an accelerating voltage of 20 KV, and mass resolution was maximized at 1600 Da. All the acquired spectra were processed using 4700 Explore^TM^ software (Applied Biosystems, Vernon Hills, IL, USA) at default settings. A combined search was performed against all updated entries from the NCBInr and Fungi MSDB sequence databases via in-house MASCOT server (v.2.3 MatrixScience, London, UK). The search parameters were: Enzyme, trypsin; Fixed modifications, carbamidomethyl (C); Variable modification, oxidation (M); Peptide mass tolerance, 40–100 ppm; Maximum missed cleavages, 2. The accepted MOWSE score threshold was inferred at *P < 0.05*. False-discovery rate (FDR) [41] for the peptide search match was calculated using a decoy database at cut-off FDR ≤ 1%.

### Availability of supporting data

Sequences dataset of *calmodulin* locus used for phylogenetic reconstruction and detail morphological descriptors for *A. terreus* can be accessed in Dryad repository at http://dx.doi.org/10.5061/dryad.590j0.

## Abbreviations

PC: Phialidic conidia; AC: Accessory conidia; BIC: Bayesian information criterion; AICc: Akaike information criterion, corrected; ML: Maximum likelihood; SEM: Scanning electron microscopy; 1-D: One dimensional SDS gel electrophoresis; Mr: Molecular mass; pI: Isoelectric pH; IEF: Isoelectric focusing; 2-D: Two dimensional SDS gel electrophoresis; CBR: Coomassie brilliant blue R250; IPGs: Immobilized pH gradient strips; MALDI-TOF/TOF MS/MS: Matrix assisted laser desorption/ionization-time of flight/time-of-flight tandem mass spectrometry.

## Competing interests

The authors declare that they have no competing interests.

## Authors’ contributions

BL conceived the experiment and performed all 1-D and 2-D analysis and first interpretation of the data. SDW performed the phialidic conidia count experiments and statistical exploration. PR assisted in the designing the experiment, guided in all gel-based analysis and participated in writing. PKB assisted in MS/MS analysis and data interpretation. WMS performed the pathogenicity test. CKS assisted in the interpreted of SEM images. NCT participated in the interpretation of the MS/MS data, and all authors wrote the manuscript and approve the final manuscript.

## Supplementary Material

Additional file 1: Figure S1 **(A)***A. terreus* forms interconnected hyphae network on potato cv. Kufri Jyoti by 96 h after inoculation hallmarked by sporulation, at 800X. **(B)** Potato cv. Kufri Jyoti showing foliar blight cause by *A. terreus*. Necrotic spot with white mycelia patch is encircled. Click here for file
