# Supplementary figures and images for: Invasion of Solanum tuberosum L. by Aspergillus terreus: a microscopic and proteomics insight on pathogenicity
Source: BMC Res Notes. 2014 Jun 10;7:350. doi: 10.1186/1756-0500-7-350 (PMC4065392; doi:10.1186/1756-0500-7-350)

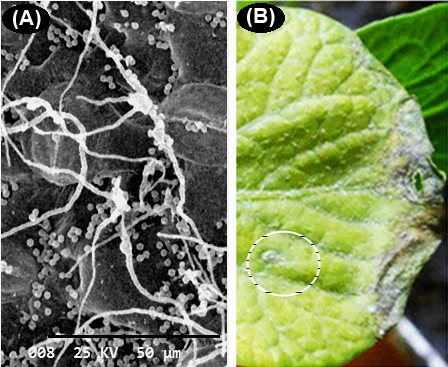

Supplement: Additional file 1: Figure S1 — (A)A. terreus forms interconnected hyphae network on potato cv. Kufri Jyoti by 96 h after inoculation hallmarked by sporulation, at 800X. (B) Potato cv. Kufri Jyoti showing foliar blight cause by A. terreus. Necrotic spot with white mycelia patch is encircled. [file 1756-0500-7-350-S1.tiff]
